# Maternal Fructose Intake Affects Transcriptome Changes and Programmed Hypertension in Offspring in Later Life

**DOI:** 10.3390/nu8120757

**Published:** 2016-11-25

**Authors:** You-Lin Tain, Julie Y. H. Chan, Chien-Ning Hsu

**Affiliations:** 1 Department of Pediatrics, Kaohsiung Chang Gung Memorial Hospital, Chang Gung University College of Medicine, Kaohsiung 833, Taiwan; tainyl@hotmail.com; 2 Institute for Translational Research in Biomedicine, Kaohsiung Chang Gung Memorial Hospital, Chang Gung University College of Medicine, Kaohsiung 833, Taiwan; jchan@cgmh.org.tw; 3 Department of Pharmacy, Kaohsiung Chang Gung Memorial Hospital, Kaohsiung 833, Taiwan; 4 School of Pharmacy, Kaohsiung Medical University, Kaohsiung 807, Taiwan

**Keywords:** developmental programming, developmental origins of health and disease (DOHaD), fructose, hypertension, kidney, next-generation sequencing, reprogramming, transcriptome

## Abstract

Hypertension originates from early-life insults by so-called “developmental origins of health and disease” (DOHaD). Studies performed in the previous few decades indicate that fructose consumption is associated with an increase in hypertension rate. It is emerging field that tends to unfold the nutrient–gene interactions of maternal high-fructose (HF) intake on the offspring which links renal programming to programmed hypertension. Reprogramming interventions counteract disturbed nutrient–gene interactions induced by maternal HF intake and exert protective effects against developmentally programmed hypertension. Here, we review the key themes on the effect of maternal HF consumption on renal transcriptome changes and programmed hypertension. We have particularly focused on the following areas: metabolic effects of fructose on hypertension and kidney disease; effects of maternal HF consumption on hypertension development in adult offspring; effects of maternal HF consumption on renal transcriptome changes; and application of reprogramming interventions to prevent maternal HF consumption-induced programmed hypertension in animal models. Provision of personalized nutrition is still a faraway goal. Therefore, there is an urgent need to understand early-life nutrient–gene interactions and to develop effective reprogramming strategies for treating hypertension and other HF consumption-related diseases.

## 1. Introduction

Fructose consumption has grown over the past several decades and its growth has been paralleled by an increase in hypertension [1,2,3]. Nutrition during pregnancy and lactation exerts long-term effects on the health of offspring. Developmental origins of health and disease (DOHaD) is an emerging branch of science that assesses the effects of these early insults on the health of offspring [4]. Adult-onset hypertension develops from nutritional insults in early life [5]. Because the developing kidney is particularly vulnerable to insults of programming in early life, renal programming plays an essential role in the developmental programming of hypertension [6]. The DOHaD concept offers a novel approach to prevent programmed hypertension through reprogramming [7].

This review provides an overview of maternal high-fructose (HF) consumption-induced gene–diet interactions in the offspring kidneys that affect programmed hypertension, with an emphasis on the following areas: metabolic effects of fructose on hypertension and the kidney; effects of maternal HF consumption on programmed hypertension; effects of maternal HF consumption on renal transcriptome changes; and application of reprogramming interventions to prevent maternal HF-induced programmed hypertension.

## 2. Metabolic Effects of Fructose on Renal Biology and Hypertension

Fructose is a monosaccharide naturally present in honey, fruits, and vegetables. In our body, fructose is endogenously produced from glucose through aldose reductase pathway and is also obtained through exogenous supply [8]. Because the food industry refines fructose and adds it to various processed foods, our fructose consumption has increased dramatically in the past few decades [2]. Most of our daily fructose comes from HF corn syrup and refined sugar (e.g., table sugar). Fructose is absorbed in the intestine through specific glucose transporters such as glucose transporter 5 (Glut 5) and Glut 2. The liver is the major site of fructose metabolism. Fructose is converted into glucose, lactate, and fatty acids [8]. Fructose metabolism differs markedly from glucose metabolism because these two sugars require different enzymes in the initial steps of metabolism. Fructose is oxidized to CO_2_ and is then converted to lactate and glucose; moreover, fructose leads to ATP depletion and uric acid production and does not induce insulin release [8].

Limited epidemiological data indicate that fructose exerts pressor effects, thus increasing blood pressure (BP) [9,10]. Although human experimental studies have reported the acute effects of dietary fructose on BP [11,12,13], its chronic effects have not been established to date. Moreover, although the kidneys are particularly sensitive to the effects of fructose, only a few epidemiological studies have examined the relationship between fructose consumption and renal disease [11]. Thus, human studies have not yet established the direct cause-and-effect relationship between excessive fructose consumption and hypertension and kidney disease. HF diets have been used to generate animal models of hypertension and kidney disease [14,15,16]. Similar to the results of human studies [17,18], results of animal studies indicate that rats fed HF diet develop various features of metabolic syndrome, including hypertriglyceridemia, insulin resistance, obesity, hyperinsulinemia, and hypertension [16,19]. Adverse effects of fructose feeding depend on the amount and duration of fructose consumption. Because rats express uricase (which degrades uric acid) and because they develop early phenotypes after exposure to high fructose concentrations, most studies on rats have been performed using diets containing 50%–60% fructose [16]. Although most studies on fructose-induced hypertension have used fructose doses amounting to ~60% of the total energy requirement [16], evidence indicates that 20% fructose diet significantly increases BP in rats after 8 months [20]. Fructose induces renal hypertrophy and tubulointerstitial disease in the rat kidneys [21]. Numerous pathways have been proposed to induce fructose-induced hypertension, including oxidative stress, increased sodium absorption, endothelial dysfunction, nitric oxide (NO) deficiency, renin-angiotensin system (RAS) activation, and sympathetic nervous system stimulation [16,22]. Fructose increases the reabsorption of salt and water in the kidneys; thus, a combination of fructose and salt exerts synergistic effect on hypertension development [23].

## 3. Effect of Maternal Fructose Consumption on Programmed Hypertension

Although numerous studies have assessed the effect of fructose on adult metabolism, limited studies have explored the effects of maternal fructose consumption on fetus and disease risk in offspring. Thus far, only a limited number of human studies have shown an association between excessive sweetened food and beverage consumption and poor pregnancy outcome [24]. Animal studies have shown that fructose alone alters fetal and offspring metabolism [24]. However, several animal studies have often used fructose as a part of diet along with sucrose, fat, and salt.

Several studies have shown that HF diet induces hypertension in adult rats, which have been well reviewed elsewhere [16,22,25]. However, limited data are available at present on the effects of maternal fructose consumption on the BP of adult offspring. Studies listed in Table 1 indicate that consumption of HF alone or as a part of diet by rodent mothers induces programmed hypertension in adult offspring [26,27,28,29,30,31,32,33]. We found that adult offspring of mothers exposed to 60% HF diet during pregnancy and lactation developed hypertension [26], which is consistent with the results of earlier studies involving fructose-fed adult rats [16,19]. “Western diet” is characterized by the high intake of high-sugar drinks, high-fat products, and excess salt. Therefore, it is important to consider the potential interactions and programmed processes between fructose, fat, and salt. Animal studies examining the combined effects of maternal fructose consumption and other key components of the Western diet (e.g., high fat and high salt) have shown their synergistic effects on the elevation of BP in adult offspring [29,30].

In adult rats, HF intake for >8 weeks induces renal damage [16]. However, our recent data indicate that rats receiving HF diet do not develop renal damage until 3 months of age. Unlike fructose-induced uric acid generation that induces oxidative stress and NO deficiency in adult rats [14,16,22], maternal HF consumption-induced programmed hypertension does not induce these abnormalities in adult offspring [27]. These data suggest that mechanisms underlying maternal HF consumption-induced programmed hypertension in offspring are different from those underlying fructose feeding-induced programmed hypertension adult rats.

## 4. HF Consumption Induces Renal Transcriptome Changes

Notably, almost entire oral fructose consumed by pregnant mother rats is converted to glucose, glycogen, fat, and lactate in the liver and is released into circulation [8]. Because fructose can be transported across the human placenta [34] and because human placenta generates endogenous fructose [35], it can be suggested that the key fetal programming process is driven by both fructose and its metabolites. Nutrigenomics has been introduced to understand existing reciprocal interactions between genes and nutrients [36]. Among different molecular nutrition approaches, transcriptomics provides information on mechanisms and physiological signals of a particular diet at a molecular level [36]. Recent advances in next-generation sequencing (NGS) allow us to monitor gene-diet interactions at a genome-wide level. The nutrigenomics approach indicates that fructose consumption leads to significant transcriptome changes in the brain of rats [37]. However, only limited studies have analyzed the transcriptome of the kidneys isolated from rodent models of maternal fructose consumption. We performed NGS by using RNA isolated from a 1-day-old offspring to analyze transcriptome changes in response to maternal HF consumption [28,38]. We found that in addition to genes associated with fructose metabolism, genes associated with other metabolic pathways such as glycolysis/gluconeogenesis, fatty acid metabolism, and insulin signaling were differential expressed (Table 2). Expression of genes encoding liver-type 6-phosphofructokinase (*Pfkl*), peroxisome proliferator-activated receptor gamma coactivator 1-α (*Ppargc1a*), glucose transporter 1 (*Slc2a1*), insulin receptor substrate 2 (*Irs2*), lactate dehydrogenase A (*Ldha*), and sterol regulatory element-binding transcription factor 1 (*Srebf1*) was upregulated in the kidneys. We also examined major organs that control BP, including the heart and brain, and observed that maternal HF consumption increased the mRNA levels of *Pfkl*, hexokinase 2 (*Hk2*), 6-phosphofructo-2-kinase/fructose-2,6-biphosphatase 3 (*Pfkfb3*), suppressor of cytokine signaling 3 (*Socs3*), NFκB inhibitor α (*Nfkbia*), *Ppargc1a*, liver glycogen phosphorylase (*Pygl*), and forkhead box protein O1 (*Foxo1*) in the heart. However, mRNA expression of only *Slc2a1* and short/branched chain specific acyl-CoA dehydrogenase (*Acadsb*) was upregulated, whereas that of *Socs3* was downregulated in the brain. Moreover, in contrast to the tightly regulated glucose metabolism in the brain, insulin signaling was perturbed in the kidneys and heart. Thus, our data suggest that different organs react differently to developmental programming, leading to organ-specific transcriptional modification of gene cascades.

**Figure 1:**
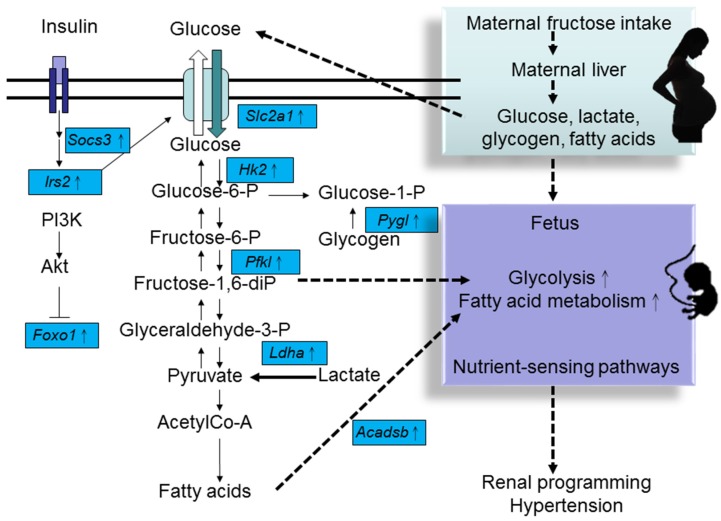
Schematic representation of changes in the expression of genes regulating glucose metabolism, fatty acid metabolism, and insulin signaling in the kidneys of offspring exposed to maternal HF diet. Solid lines with arrowheads indicate known signaling events and interactions between glucose metabolism, fatty acid metabolism, and insulin signaling. Dashed lines with arrowheads denote proposed mechanisms contributing to maternal HF consumption-induced programmed hypertension. Solid square boxes indicate DEGs identified by next-generation sequencing (NGS).

Our data showed that maternal HF consumption elicited different metabolic pathways in the developing kidney and heart. A schematic representation of maternal HF consumption-induced transcriptome changes in fructose metabolism, glycolysis/gluconeogenesis, fatty acid metabolism, and insulin signaling is shown in Figure 1. Fructose and related sugars, amino acids, and fatty acids are important cellular nutrients. Specific nutrients function as signaling molecules that transmit and translate dietary signals into changes in gene expression through appropriate sensing mechanisms (also known as nutrient-sensing pathway) [39]. Transcription factors are the main agents through which nutrients influence gene expression. Nuclear receptor superfamily of transcription factors is the most important group of nutrient sensors. For example, peroxisome proliferator-activated receptors (PPARs) interact with other nutrient-sensing signals to trigger renal programming and hypertension in response to maternal nutritional insults [40]. Our NGS data suggest that the nutrient-sensing pathway is crucial for the response of different organs of offspring to maternal HF consumption for programming differential phenotypes of metabolic syndrome, including hypertension.

Our NGS data identified 10 significantly related Kyoto Encyclopedia of Genes and Genomes (KEGG) pathways shared by 3 different developmental windows in the kidneys of offspring exposed to maternal HF diet [28]. These KEGG pathways include complement and coagulation cascades; PPAR signaling; hematopoietic cell lineage; circadian rhythm; fatty acid metabolism; valine, leucine and isoleucine degradation; cell adhesion molecules; adipocytokine signaling pathway; arachidonic acid metabolism; and butanoate metabolism. Of these, the complement and coagulation cascade pathway is significantly regulated by maternal HF consumption, which is consistent with the results of a previous study involving a rat model of intrauterine growth retardation [41]. Arachidonic acid metabolism is another significant maternal HF consumption-related KEGG pathway. Arachidonic acid is metabolized by cytochrome P450, cyclooxygenase, or lipoxygenase to prostaglandins and related compounds [42]. We recently reported that arachidonic acid metabolites are the key components involved in hypertension development in various animal models [43].

In total, 20 DEGs in the kidneys of 1-day-old offspring exposed to maternal HF diet are associated with BP regulation [24]. Of these, *Adra2b*, *Bdkrb2*, *Col1a2*, *Hmox1*, *Ptgs2*, and *Tbxa2r* are associated with endothelium-derived hyperpolarizing factors (EDHFs). Because EDHFs play a crucial role in maintaining maternal and fetal circulation, our data suggest that early-life fructose exposure prevents interrelated EDHFs from adapting during nephrogenesis, leading to programmed hypertension in later life. Furthermore, our NGS data suggest that nutrigenomics approach can identify renal programming-associated genes and pathways that can be used as potential therapeutic targets for prevent maternal HF consumption-induced programmed hypertension in adult offspring.

Epigenetic regulation may induce programmed hypertension [6,7]. We used a maternal HF consumption model to analyze five groups of epigenetic regulators in the kidneys of 1-day-old offspring. Of these, expression of seven genes, namely, *Dnmt3l*, *Hdac9*, *Hdac11*, *Chd2*, *Brdt*, *Brwd1*, and *Myst2*, were found to be significantly regulated [28]. However, additional nutrigenomics studies are needed to determine whether fructose-induced epigenetic regulation, including DNA methylation, histone acetylation, and microRNA interference, is involved in maternal HF consumption-induced programmed hypertension.

## 5. Reprogramming Strategy to Prevent Maternal HF Consumption-Induced Programmed Hypertension

Several intervention strategies, including taurine, arginine, resveratrol, grape-derived polyphenols, sardine protein, vitamin E, and α-lipoic acid, have been used to prevent the adverse metabolic effects of excess fructose consumption in adults [44]. However, none of these strategies has been examined as a candidate reprogramming strategy for preventing maternal HF consumption-induced programmed hypertension.

Our data suggest that programmed processes promoting maternal HF consumption-induced programmed hypertension are different from those promoting fructose feeding-induced programmed hypertension in adult rats. Different mechanisms have been proposed to induce programmed hypertension, such as epigenetic regulation, glucocorticoid effects, RAS and sodium transporter alterations, oxidative stress, and nephron number reduction; these mechanisms can serve as potential targets for preventing maternal HF consumption-induced programmed hypertension [7,45]. The renal transcriptome is greatly altered in the adult offspring of various models of programmed hypertension [39,40]. We prevented hypertension development in adult offspring exposed to maternal HF diet by using three deprogramming approaches, namely, melatonin [27], soluble epoxide hydrolase (SEH) inhibitor [28], and renin inhibitor aliskiren.

Most reprogramming strategies have focused on restoring the balance of NO and reactive oxygen species (ROS) to prevent hypertension [7]. Melatonin is an endogenously produced indoleamine that exerts pleiotropic effects, including antioxidant effects [46]. We observed that maternal melatonin treatment prevented HF consumption-induced programmed hypertension and increased NO levels in the offspring kidneys [27]. Thus, reprogramming strategies that restore the NO–ROS balance can be applied in a broad range of prohypertensive developmental conditions.

Our NGS data indicate that the arachidonic acid metabolism pathway is involved in maternal HF consumption-induced renal programming and programmed hypertension [27,28]. Analysis by using two models of programmed hypertension indicated *Ephx2* expression and SEH (encoded by *Ephx2*) activity played a direct role in renal programming [43]. Our recent studies indicate that early postnatal treatment targeting the arachidonic acid metabolism pathway by using an SEH inhibitor 12-(3-adamantan-1-yl-ureido)-dodecanoic acid (AUDA) ameliorates hypertension in both maternal HF consumption-induced and prenatal dexamethasone-induced hypertension models [29,47]. Moreover, AUDA is effective in reprogramming BP in female spontaneously hypertensive rats (SHRs) but not in male SHRs [48]. Thus, reprogramming interventions for preventing hypertension may affect pathways that are common to nutrition and genetic models. However, it would be interesting to see whether SEH inhibition also prevents programmed hypertension in other models of nutritional programming.

RAS plays an essential role in BP control and nephrogenesis. Blockade of RAS with an angiotensin-converting enzyme inhibitor captopril, angiotensin receptor blocker losartan, or renin inhibitor aliskiren in young offspring from age 2 to 4 weeks of various animal models of hypertension counteracts programming effects [49,50,51]. We recently found that aliskiren administration during early postnatal life prevented maternal HF consumption-induced programmed hypertension in adult offspring of both the sexes [30]. We also observed that maternal HF consumption induced higher changes in the renal transcriptome of female rats than in that of male rats at 1 week of age [29]. Because sex differences exist in experimental models and human studies of hypertension [52], future studies should be aimed at identifying fundamental sex-specific mechanisms to provide a novel reprogramming strategy for achieving maximal optimization in both the sexes.

## 6. Conclusions

Diet is a major environmental factor in gene–environment interactions underlying the DOHaD concept. Maternal nutrition and its association with nutrient–gene interactions remains a challenging area of research. Although results obtained using animal models indicate that maternal HF consumption plays a role in the developmental programming of hypertension, early-life fructose–gene interactions in humans might be more complex and multifactorial. However, results of animal studies indicate that downstream pathways are largely reprogrammable irrespective of their upstream stimuli. This is fortunate because identification of upstream stimuli is often difficult in humans with programmed hypertension. Applications of newly developed high-throughput tools in nutrigenomics will allow us to identify genes or metabolites that are altered during prehypertension and will help in characterizing pathways regulated by dietary fructose. These tools can also help in developing early diagnostic methods and effective reprogramming strategies for treating HF diet-related diseases such as hypertension and metabolic syndrome. These new findings should be confirmed in further studies to develop personalized nutrition for health promotion and disease prevention.

## Figures and Tables

**Table 1:** Maternal high-fructose (HF) consumption exerts programming effects on blood pressure (BP) in rodent models.

Types of Fructose Intake	Strain	Programming Effects	Age at Which the Effects Were Measured	References
10% *w*/*v* fructose plus 4% NaCl in drinking water 28 days before conception and throughout gestation and lactation	Male Sprague–Dawley rats	↑ systolic BP, ↑ mean arterial BP	At 9 weeks of age	[26]
60% HF diet throughout pregnancy and lactation	Male Sprague–Dawley rats	↑ systolic BP, ↑ mean arterial BP	At 12 weeks of age	[27,28,29]
60% HF diet throughout pregnancy and lactation	Male and female Sprague–Dawley rats	↑ systolic BP	At 12 weeks of age	[30]
60% HF diet throughout pregnancy and lactation plus 1% NaCl in drinking water from weaning to 3 months of age	Male Sprague–Dawley rats	↑ systolic BP, ↑ mean arterial BP; postnatal high-salt aggravates prenatal HF-induced programmed hypertension	At 12 weeks of age	[31]
56.7% HF/high-fat diet throughout pregnancy and lactation	Male Sprague–Dawley rats	↑ mean arterial BP	At 16 weeks of age	[32]
10% *w*/*v* fructose in drinking water throughout pregnancy and lactation	C57BL/6J mice	↑ mean arterial BP, obesity, metabolic dysfunction	At 12 months of age	[33]

Studies have been tabulated according to the age at which the effects were measured.

**Table 2:** Changes in the expression of shared differential expressed genes (DEGs) associated with fructose metabolism in the kidneys, brain, and heart of offspring exposed to maternal HF diet at 1 day of age.

Gene ID	Symbol	Kidney	Brain	Heart
Fructose and mannose metabolism				
ENSRNOG00000001214	*Pfkl*	**2.3**	1.5	**2.2**
ENSRNOG00000006116	*Hk2*	1.8	ND	**2.1**
ENSRNOG00000018911	*Pfkfb3*	1.8	ND	**4.5**
Adipocytokine signaling pathway				
ENSRNOG00000002946	*Socs3*	1.6	**0.5**	**3.9**
ENSRNOG00000007390	*Nfkbia*	1.9	1.9	**3.5**
ENSRNOG00000004473	*Ppargc1a*	**2.3**	1.6	**2.7**
ENSRNOG00000007284	*Slc2a1*	**3.0**	**2.3**	ND
ENSRNOG00000023509	*Irs2*	**2.1**	ND	1.6
Glycolysis/Gluconeogenesis				
ENSRNOG00000001214	*Pfkl*	**2.3**	1.5	**2.2**
ENSRNOG00000006116	*Hk2*	1.8	ND	**2.1**
ENSRNOG00000013009	*Ldha*	**2.2**	ND	1.6
Fatty acid metabolism				
ENSRNOG00000020624	*Acadsb*	1.9	**2.0**	ND
Insulin signaling pathway				
ENSRNOG00000002946	*Socs3*	1.6	**0.5**	**3.9**
ENSRNOG00000004473	*Ppargc1a*	**2.3**	1.6	**2.7**
ENSRNOG00000006388	*Pygl*	1.9	ND	**3.2**
ENSRNOG00000006116	*Hk2*	1.8	ND	**2.1**
ENSRNOG00000023509	*Irs2*	**2.1**	ND	1.6
ENSRNOG00000003463	*Srebf1*	**2.1**	ND	1.6
ENSRNOG00000013397	*Foxo1*	1.8	ND	**2.2**

Gene expression was quantified as reads per kilobase of exon per million mapped reads (RPKM). Genes that changed by RPKM of >0.3 and ≥2-fold differences between HF vs. control. Significant results are highlighted in bold. ND, not detectable.
